# Cluster headache and arachnoid cyst

**DOI:** 10.1186/2193-1801-2-4

**Published:** 2013-01-10

**Authors:** Bengt Edvardsson, Staffan Persson

**Affiliations:** Department of Neurology, Faculty of Medicine, Skane University Hospital, S-221 85 Lund, Sweden

**Keywords:** Cluster headache, Arachnoid cyst, Neuroimaging, Secondary, Symptomatic, Magnetic resonance imaging, Computer tomography

## Abstract

**Background:**

Cluster headache is a primary headache by definition not caused by any known underlying structural pathology. However, symptomatic cases have been described, e.g. tumours, particularly pituitary adenomas, malformations, and infections/inflammations. The evaluation of cluster headache is an issue unresolved.

**Case description:**

We present a case of a 43-year-old patient who presented with a 2-month history of side-locked attacks of pain located in the left orbit. He satisfied the revised International Classification of Headache Disorders criteria for cluster headache. His medical and family histories were unremarkable. There was no history of headache. A diagnosis of cluster headache was made. The patient responded to symptomatic treatment. Computer tomography and enhanced magnetic resonance imaging after 1 month displayed a supra- and intrasellar arachnoid cyst with mass effect on adjacent structures. After operation, the headache attacks resolved completely.

**Discussion and evaluation:**

Although we cannot exclude an unintentional comorbidity, in our opinion, the co-occurrence of an arachnoid cyst with mass effect with unilateral headache, in a hitherto headache-free man, points toward the fact that in this case the CH was caused or triggered by the AC. The headache attacks resolved completely after the operation and the patient also remained headache free at the follow-up. The response of the headache to sumatriptan and other typical CH medications does not exclude a secondary form. Symptomatic CHs responsive to this therapy have been described. Associated cranial lesions such as tumours have been reported in CH patients and the attacks may be clinically indistinguishable from the primary form.

**Conclusions:**

Neuroimaging, preferably contrast-enhanced magnetic resonance imaging should always be considered in patients with cluster headache despite normal neurological examination. Late-onset cluster headache represents a condition that requires careful evaluation. Supra- and intrasellar arachnoid cyst can present as cluster headache.

## Introduction

Cluster headache (CH) is a primary headache, by definition not caused by any underlying structural pathology and belonging to the group of trigeminal-autonomic cephalalgias (Headache Subcommittee of the International Headache Society 2004). CH is the most frequent syndrome in this group. Symptomatic cases of CH have been described, e.g. tumours, particularly pituitary adenomas, malformations, and infections/inflammations (Cittadini & Matharu 2009).

Arachnoid cysts (AC) are extra-parenchymal and intra-arachnoidal collections of fluid with a composition similar to that of cerebrospinal fluid (CSF). Headaches are the most frequent complaint of patient with an AC. However, the origin of headaches is manifold and in many patients with headaches, AC must be regarded as an incidental finding (Westermaier et al. 2012).

The question whether patients with CH should undergo neuroimaging to exclude a causal underlying structural lesion is unresolved. AC causing typical CH has not been described. We report a patient with a typical CH in the setting of a supra-and intrasellar arachnoid cyst.

## Case report

**Figure 1:**
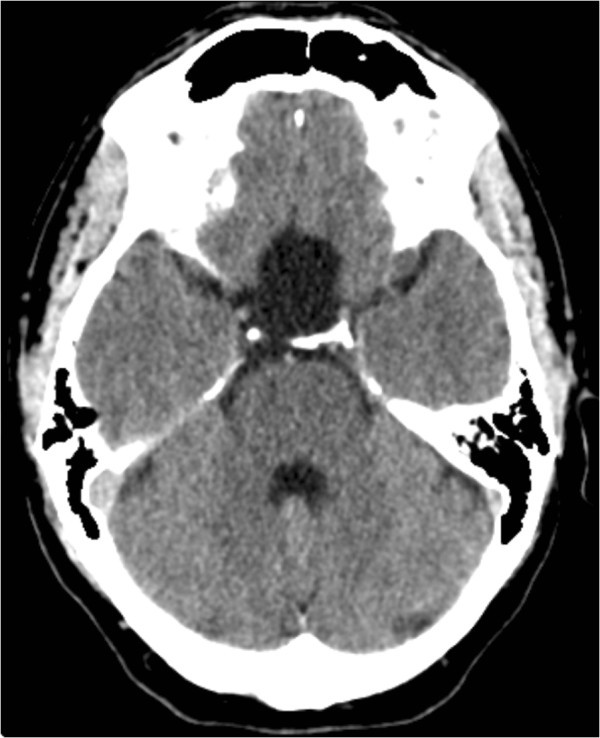
**CT of the brain demonstrating a large supra- and intrasellar arachnoid cyst with mass effect on adjacent structures.**

**Figure 2:**
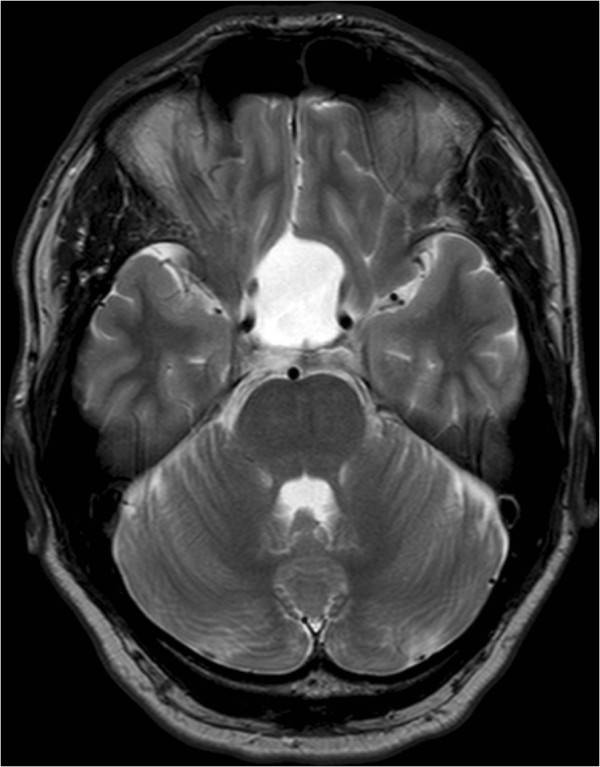
**MRI, T2-weighted imaging demonstrating a large supra- and intrasellar arachnoid cyst with mass effect on adjacent structures.**

A 43-year-old man presented with a 2-month history of side-locked attacks of excruciatingly severe stabbing and boring left-sided pain located in the orbit. The attacks were associated with nasal obstruction, conjunctival injection, and restlessness and migrainous features such as nausea and photophobia/phonophobia. No continuous background pain was identified. The duration of the attacks was about 30 min and the frequency 4 to 5 per 24 h, 3 to 4 days a week and they also occurred during the night. There was no history of headache. His medical and family history was otherwise unremarkable. He was not on any medications and used no drugs. Vital signs, physical examination, and neurological examination were normal. Laboratory testing was normal. He satisfied the revised International Classification of Headache Disorders criteria for CH. A diagnosis of CH was made and subcutaneous sumatriptan as well as oral sumatriptan were prescribed. A prophylactic treatment with steroids and verapamil was suggested but the patient preferred symptomatic medication instead of using a prophylactic drug for CH. He responded to the treatment with relief within 15 to 20 min. A follow-up was planned. As the headache attacks continued, the patient was hospitalized after about 1 month. At admission, the neurological examination was normal. He was on the following medication: subcutaneous/oral sumatriptan when required. A CT scan of the head displayed a supra- and intrasellar arachnoid cyst with mass effect (Figure 1). An enhanced magnetic resonance imaging (MRI) was ordered in order to further evaluate the lesion. It confirmed the diagnosis of a supra- and intrasellar arachnoid cyst with mass effect on adjacent structures (Figure 2). Operation (craniotomy with cyst fenestration) and histopathological examination verified the diagnosis of AC. The headache attacks resolved completely after the surgery. He remained headache free and had not experienced any headache attacks at follow-up after 4 months.

## Discussion

The case study highlights a patient with CH responding to treatment. Evaluation revealed a supra- and intrasellar arachnoid cyst with mass effect on adjacent structures. The patient satisfied the revised International Classification of Headache Disorders criteria for CH (Headache Subcommittee of the International Headache Society 2004).

Although we cannot exclude an unintentional comorbidity, in our opinion, the co-occurrence of an AC with mass effect with unilateral headache, in a hitherto headache-free man, points toward the fact that in this case the CH was caused or triggered by the AC. The headache attacks resolved completely after the operation and the patient also remained headache free at the follow-up.

The response of the headache to sumatriptan and other typical CH medications does not exclude a secondary form. Symptomatic CHs responsive to this therapy have been described (Testa et al. 2008; Ad Hoc Committee on Classification of Headache 1962). Associated cranial lesions such as tumours have been reported in CH patients and the attacks may be clinically indistinguishable from the primary form (Ad Hoc Committee on Classification of Headache 1962; Favier et al. 2007).

The pathophysiology of CH is not well known. The most widely accepted theory is that primary CH is characterized by hypothalamic activation with secondary activation of the trigeminal-autonomic reflex, probably by a trigeminal-hypothalamic pathway (Cittadini & Matharu 2009).

AC develop within the arachnoid membrane because of splitting or duplication of this membrane (Westermaier et al. 2012). Many AC are asymptomatic. Headache is related to AC producing increased intracranial pressure (ICP) and often is combined with other symptoms of increased ICP (Olesen et al. 2006). The clinical symptoms are determined by the location of the cyst (Westermaier et al. 2012).

Most cases of symptomatic CH reported have had sellar/parasellar abnormality as well as in this case. The sympathetic, parasympathetic and sensory fibers of the trigeminal nerve gather as a plexus in the sinus cavernosus/hypophyseal region. Thus, nerves in this region appear to be of importance to produce symptoms of CH (Olesen et al. 2006).

The exact pathophysiology in this CH case is unknown and one can only speculate whether an intermittent elevation of ICP might trigger or cause a disturbed local nerve function in the sinus cavernosus/hypophyseal region. (Wilbrink et al. 2009) suggest that a structural lesion may cause autonomic imbalance, resulting in periodic fluctuations in the activity of the autonomic nervous system, ultimately leading to an attack-wise presentation of the symptoms. Differences in the individual threshold for triggering the parasympathetic trigeminal reflexes may also play a role (Straube et al. 2007).

(Mainardi et al. 2010) found in their review (cases published from 1975 to 2008) of 76 patients that vascular pathologies, e.g. intracranial aneurysms and dural fistulas were the first cause of secondary CH, followed by tumours. The most frequent tumoural pathology was pituitary adenomas (prolactinomas), followed by meningiomas and carcinomas of the paranasal structures. No AC were found.

Attempts have been made to define red flags indicating a secondary cause when cluster-like headache appears for the first time (Mainardi et al. 2010). Compared with primary CH, secondary CH presents at an older age (about 42 y). A late onset represents a condition that requires careful evaluation (Mainardi et al. 2010). The authors of that study also emphasize in their report that, at first observation, 50% of patients with secondary CH presented as cases fulfilling the criteria for CH, perfectly mimicking CH. Therefore, the likelihood that a secondary cause is responsible for a clinical picture mimicking a primary CH, albeit low, should always be considered (Mainardi et al. 2010).

This opinion is in accordance with the reviews by (Favier et al. 2007) and by, (Wilbrink et al.^2009^) which recommend neuroimaging in all patients with trigeminal-autonomic cephalalgias. MRI is the preferred procedure for imaging in CH cases because of its greater sensitivity to vascular disease, tumour, demyelinating disease, and infections/inflammations (Wilbrink et al. 2009; Mainardi et al. 2010).

To our knowledge, this is the first report showing an association between CH and an AC. CH might be the presenting symptom of an AC even in typical forms of that headache. Neuroimaging, preferably contrast-enhanced magnetic resonance imaging should always be considered in patients with CH. Late-onset CH represents a condition that requires careful evaluation.

The principal author takes full responsibility for the data presented in this study, analysis of the data, conclusions, and conduct of the research. The principal author had full access to those data and has maintained the right to publish any and all data independent of any third party.

Concerning approval of human studies by the appropriate ethics committee and therefore performed in accordance with the ethical standards laid down in the 1964 Declaration of Helsinki: In this case this is not appreciable.
